# Renaclic for Chronic Kidney Disease Self-Management: Formative User-Centered Co-Design Study

**DOI:** 10.2196/80680

**Published:** 2026-09-10

**Authors:** Julie Mataranga, Fiona Puppo Capodano, Josue Srougbo, Joris Galland, Adrien Ugon, Sylvia Pelayo, Corinne Isnard Bagnis

**Affiliations:** 1 Academic Digital Medical Hub, Paris, France; 2 Institut du Cerveau, Paris, France; 3 ESIEE Paris, Noisy le Grand, France; 4 Centre d'Investigation Clinique - Innovation Technologique, Lille, France; 5 Assistance Publique – Hôpitaux de Paris, 47-83 boulevard de l'hôpital, Paris, 75013, France, 1 0142177227

**Keywords:** chronic kidney disease, digital health, user-centered design, co-design, patient education, nephrology

## Abstract

**Background:**

Chronic kidney disease (CKD) affects over 850 million individuals worldwide and requires sustained patient education, shared decision-making, and self-management support. Digital health platforms may facilitate these needs; however, many lack comprehensive, user-centered design and fail to address practical and psychosocial patient concerns.

**Objective:**

This study aimed to describe the formative co-design of Renaclic, a digital platform developed with patients and health care professionals (HCPs) to support CKD education and self-management in the French health care context.

**Methods:**

A three-stage user-centered design (UCD) study was conducted between April and August 2021. Stage 1 included benchmarking of 10 French-language CKD platforms and semistructured interviews with 8 stakeholders (3 HCPs: 1 nephrologist, 1 general practitioner, 1 nurse; 5 patients: 2 novice and 3 expert users). Stage 2 consisted of two virtual co-design focus groups incorporating card-sorting and interface design activities. Stage 3 translated identified needs into structured content categories and a low-fidelity prototype. Qualitative data were analyzed thematically.

**Results:**

Benchmarking revealed fragmented educational content, limited practical guidance on home dialysis logistics, insufficient coverage of lifestyle and psychosocial concerns, and minimal interactive features. Interviews identified five main themes: need for reliable centralized information, anxiety during care transitions, demand for practical lifestyle guidance, importance of reassuring language, and value of moderated peer exchange. More than 30 thematic content items were generated and organized through card-sorting into five consolidated content categories that structured the platform’s information architecture. Co-design sessions informed mobile accessibility, simplified navigation pathways, and integration of educational, symptom-tracking, and community-oriented functionalities.

**Conclusions:**

This formative study demonstrates how participatory UCD with diverse CKD stakeholders can generate a structured, context-sensitive digital platform framework. Renaclic provides a replicable model for early-stage co-design of digital health interventions in chronic disease management. Future research will evaluate usability, engagement, and implementation in real-world settings.

## Introduction

Chronic kidney disease (CKD) affects more than 850 million individuals worldwide and represents a major public health challenge due to its progressive nature, high cardiovascular comorbidity burden, and impact on quality of life [1-3]. International guidelines emphasize the importance of patient education, shared decision-making, and early preparation for kidney replacement therapy as core components of CKD management [2]. However, patients frequently report fragmented access to information, limited coordination between care providers, and insufficient practical guidance to navigate complex treatment pathways [3].

Digital health interventions have emerged as promising tools to support chronic disease self-management, including CKD [4,5]. Recent systematic reviews suggest that digital platforms may improve knowledge acquisition, treatment preparedness, and patient engagement, provided they are usable, accessible, and contextually relevant [6,7]. However, many existing CKD platforms remain information-centered rather than user-centered, often lacking integration of psychosocial needs, practical logistics (eg, home dialysis management), and moderated peer interaction.

User-centered design (UCD), as formalized in ISO 9241‐210, promotes iterative involvement of end users throughout development to ensure alignment with real-world contexts and usability requirements [8]. Participatory and co-design approaches in digital health have been increasingly advocated to address gaps between technological innovation and implementation success [9,10]. In populations with CKD, specifically, digital literacy disparities represent a critical barrier to equitable access to digital resources [11,12]. Moreover, successful adoption of digital tools depends not only on patient usability but also on health care professional acceptability, workflow integration, and organizational readiness [13].

Despite growing interest in digital nephrology, few studies have described in detail the formative co-design processes underpinning the development of CKD-specific digital platforms in the European context. Transparent reporting of early-stage development is essential to improve reproducibility, implementation readiness, and future evaluation.

This study describes the formative development of Renaclic, a digital platform designed to support CKD education and self-management within the French health care system. The objective was not to evaluate clinical effectiveness but to co-design a platform grounded in patient and professional needs using a structured, theory-informed user-centered design approach.

## Methods

### Study Design

We conducted a formative UCD study guided by principles outlined in ISO 9241‐210 for human-centered design of interactive systems [8]. The process comprised three iterative stages commonly used in digital health development: (1) benchmarking and needs analysis, (2) co-design focus groups, and (3) platform co-development [9,10]. This staged approach supports progressive translation of user needs into functional design specifications.

### Ethical Considerations

This study involved human participants and followed principles of the Declaration of Helsinki. Ethical review was sought in accordance with institutional policies. As the study involved formative co-design interviews without collection of sensitive clinical data or intervention, it was classified as minimal risk.

All participants received written information about the study objectives and provided informed consent prior to participation.

Audio recordings and transcripts were deidentified prior to analysis. Data were stored on secure institutional servers with restricted access to the research team.

No financial compensation was provided to participants.

### Participants and Recruitment Procedures

Participants were recruited between April and May 2021 within a French academic nephrology setting (Pitié-Salpêtrière Hospital, AP-HP) and through existing professional and patient networks involved in CKD care.

Health care professionals (nephrologist, general practitioner, nurse) were identified through professional collaboration networks and invited directly by email. Inclusion criteria were active involvement in CKD management and willingness to participate in a virtual interview.

Patients were recruited through outpatient nephrology consultations and through patient association networks. Eligibility criteria included age ≥18 years, diagnosis of chronic kidney disease at any stage, and ability to participate in a virtual interview conducted in French.

All participants were informed about the study objectives and provided written informed consent prior to participation.

A total of 8 stakeholders participated in the needs analysis stage: 3 health care professionals (1 nephrologist, 1 general practitioner, and 1 nurse) and 5 adults living with CKD (2 novice users and 3 expert patients involved in patient associations or patient-partner programs).

Purposive sampling was used to ensure diversity in CKD trajectories (predialysis, dialysis, transplantation pathway), age groups, and levels of digital experience.

Patients were categorized as novice users (limited use of digital health tools and no formal engagement in patient organizations) or expert users (regular use of digital tools and/or involvement in patient associations or patient-partner initiatives).

Digital literacy was assessed pragmatically through self-reported digital use and ability to participate in virtual activities. No validated digital literacy scale was used, which is acknowledged as a limitation.

Participation was voluntary, and no financial compensation was provided. Recruitment continued until no substantially new themes emerged within stakeholder categories.

### Stage 1: Benchmarking and Needs Analysis

Ten French-language CKD platforms (FranceRein, Physidia, Renif, Diaverum, Nephrocare, Fondation du Rein Aura, Aurad, Mobidy, Sfndt, and related institutional resources) were reviewed using a qualitative evaluation grid covering content scope, navigation, accessibility, interactivity, and mobile compatibility. Semistructured interviews with patients and health care professionals explored unmet needs and expectations. Interviews were audio-recorded, transcribed verbatim, and analyzed thematically.

### Stage 2: Co-Design Focus Groups

**Figure 1.**
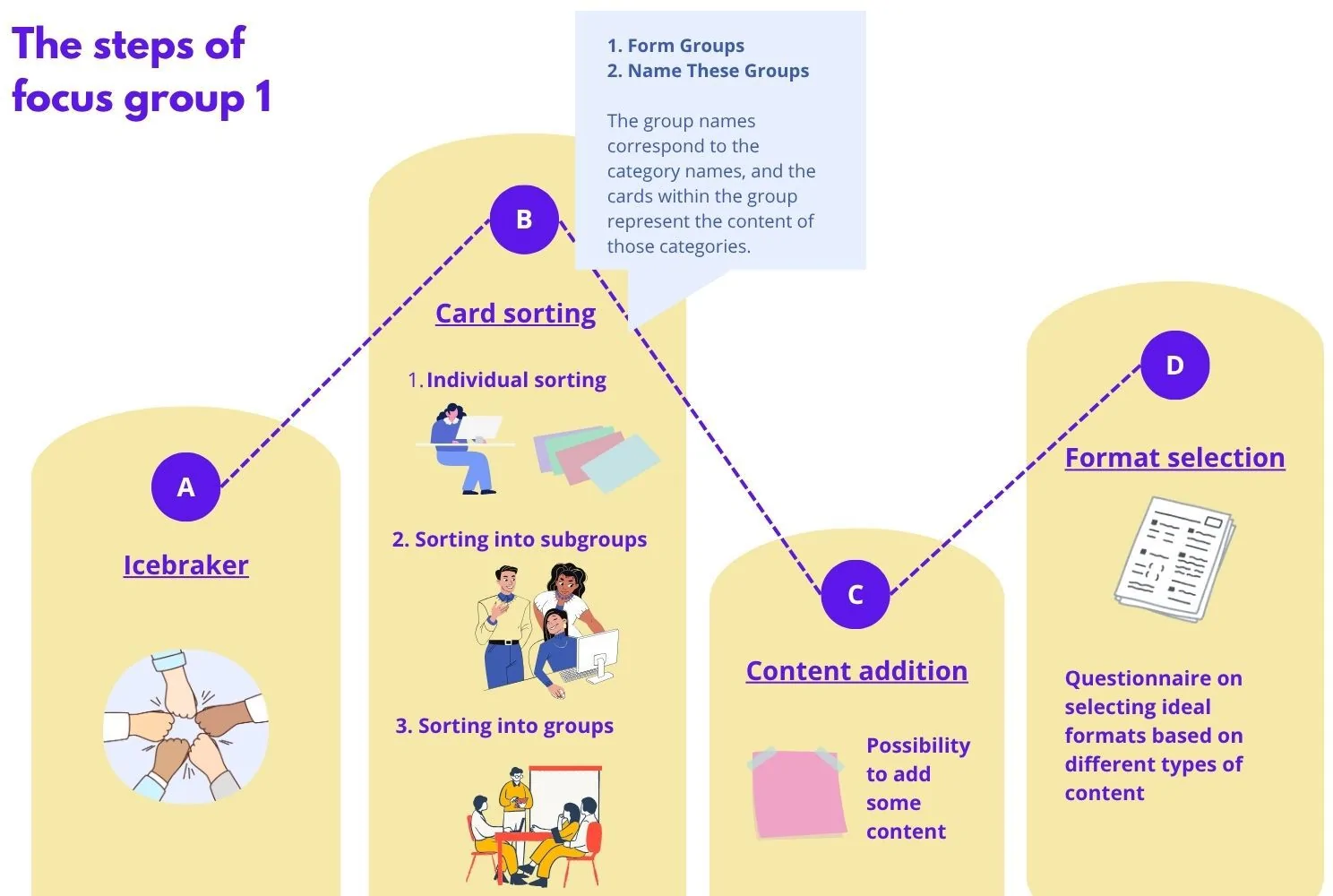
Card-sorting process conducted during Focus Group 1 in a formative user-centered design study of the Renaclic CKD platform (France, 2021).

Two virtual focus groups were conducted. Focus Group 1 used a card-sorting methodology to elicit users’ mental models and organize content categories (Figure 1).

User-centered design activity involving patients living with chronic kidney disease and health care professionals to organize more than 30 thematic content items into structured information categories, in accordance with ISO 9241‐210 human-centered design principles [8,10].

**Figure 2.**
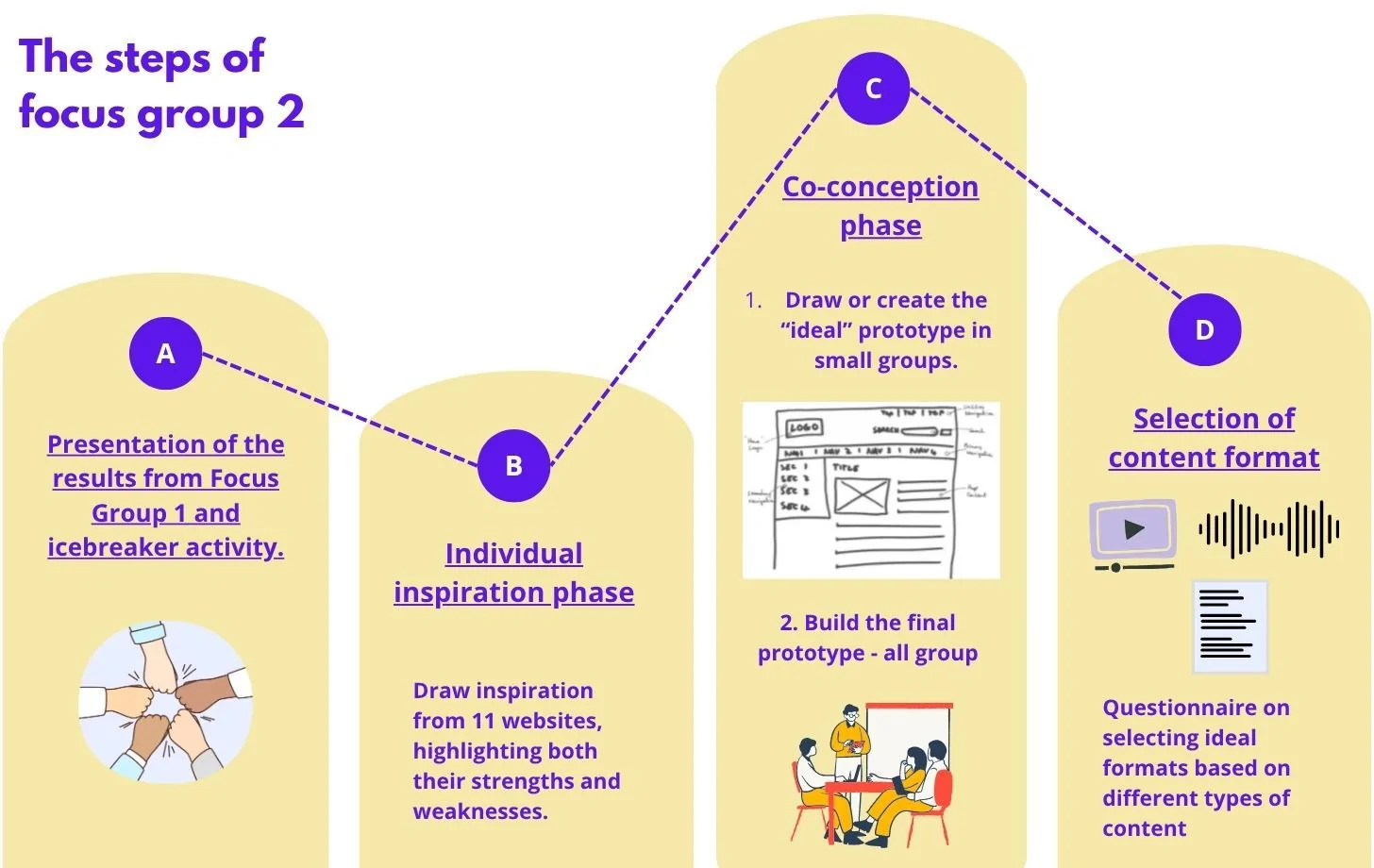
Interface co-design process conducted during Focus Group 2 in the formative development of the Renaclic CKD platform (France, 2021).

Card sorting is a recognized information-architecture technique in UCD [10]. Focus Group 2 explored interface preferences using reference websites and collaborative design tools (Figure 2).

Virtual participatory design session involving patients with CKD and health care professionals to define navigation pathways, visual hierarchy, and usability preferences following user-centered design principles [8].

**Figure 3.**
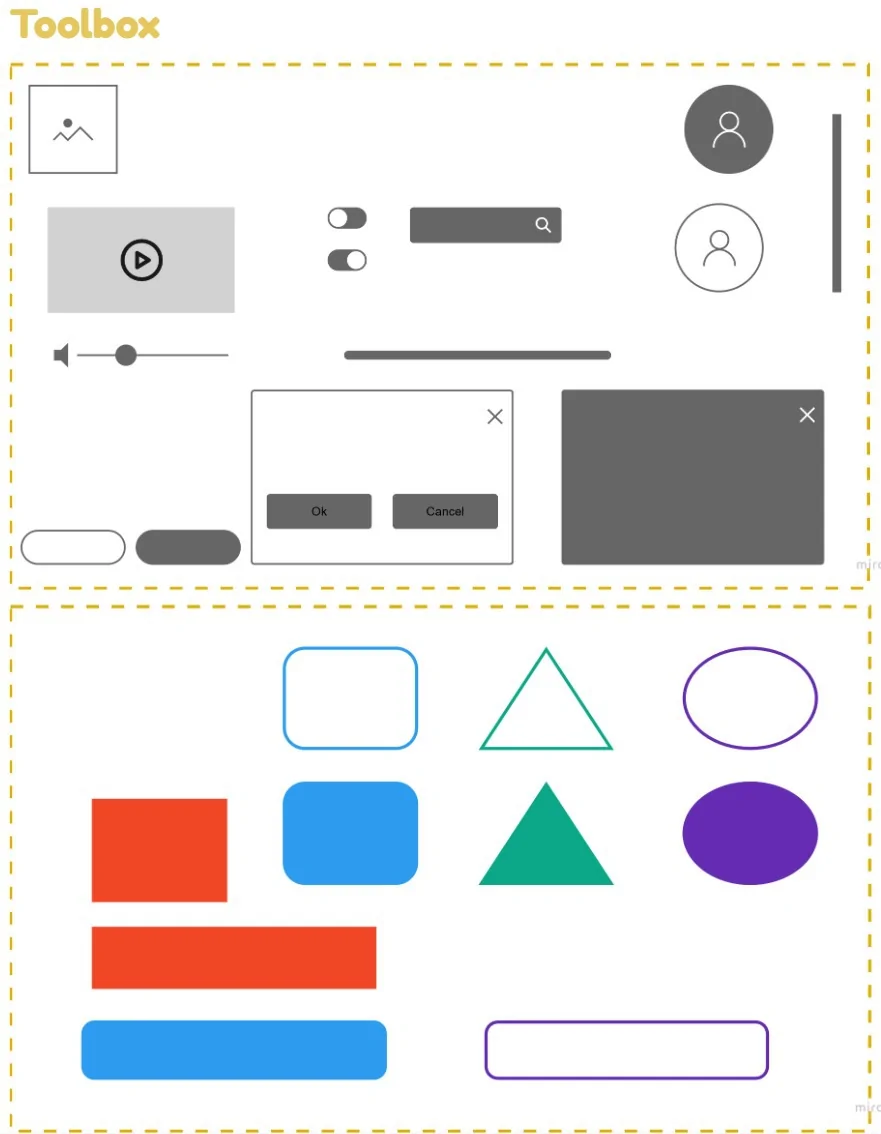
Collaborative whiteboard tools (Miro platform) used during virtual co-design sessions in the formative Renaclic study (France, 2021).

Collaborative whiteboard tools were used to support participatory design activities (Figure 3).

Example of digital participatory design environment supporting structured content organization and interface development with patients with CKD and health care professionals [9,10].

### Stage 3: Co-Design Outputs and Platform Prototype Co-Development

**Figure 4.**
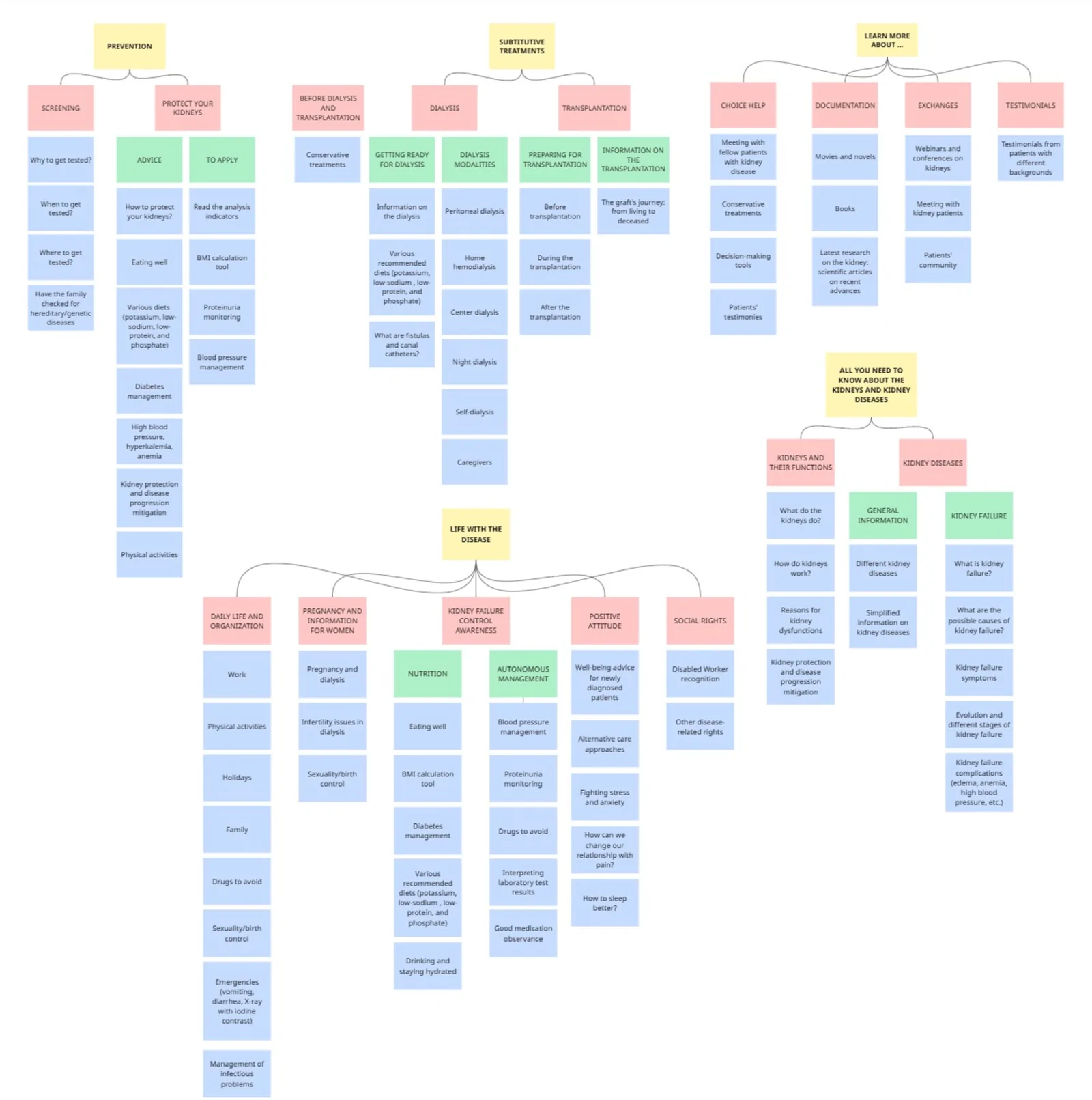
Final content architecture resulting from card-sorting activities in the formative Renaclic co-design study (France, 2021).

Outputs from the co-design activities were translated into platform specifications by a multidisciplinary team including patients, clinicians, ergonomists, and developers. The card-sorting activity resulted in five consolidated content categories that structured the final information architecture (Figure 4).

**Figure 5.**
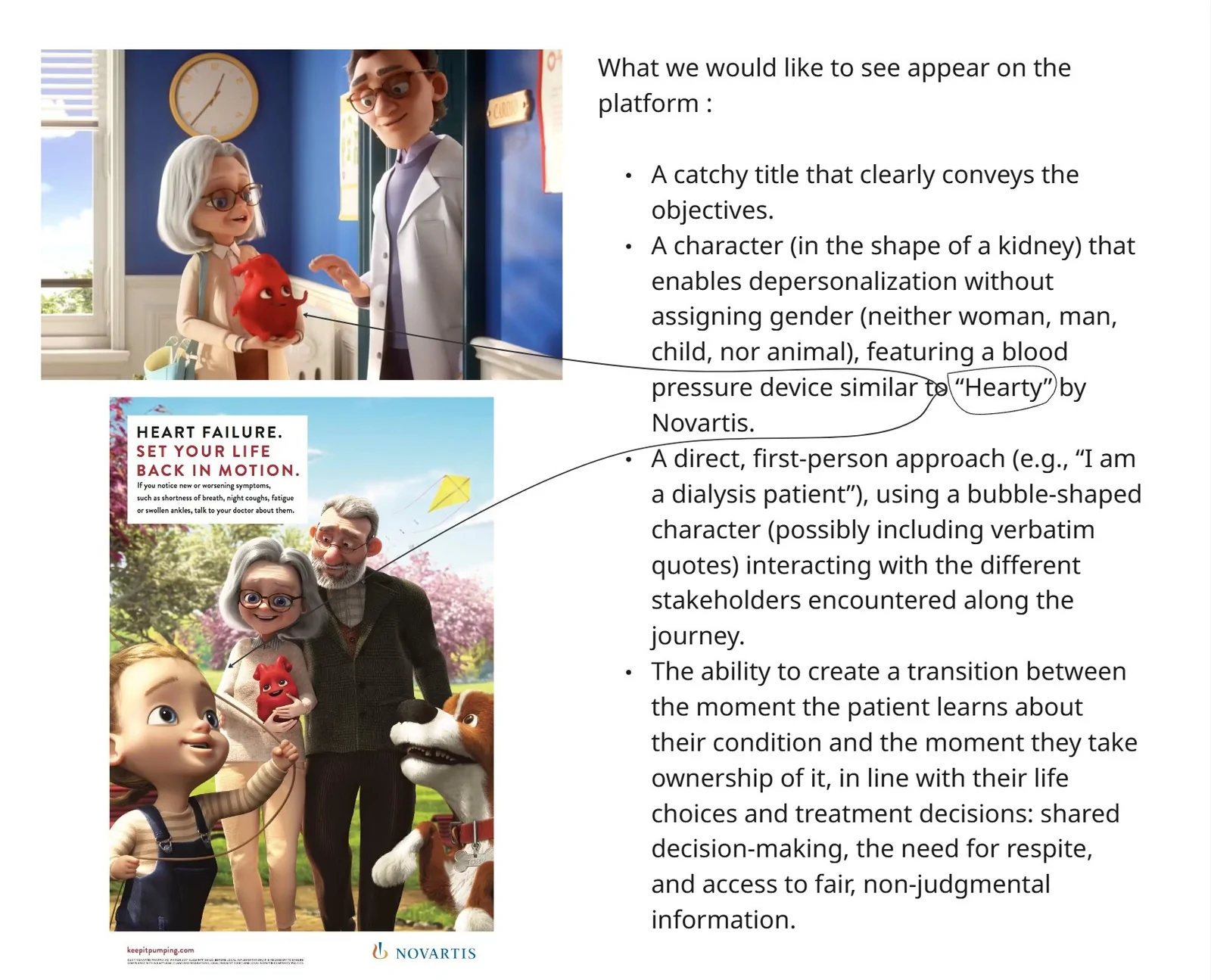
Low-fidelity prototype mockup developed during the formative co-design phase of the Renaclic CKD platform (France, 2021). CKD:

**Figure 6.**
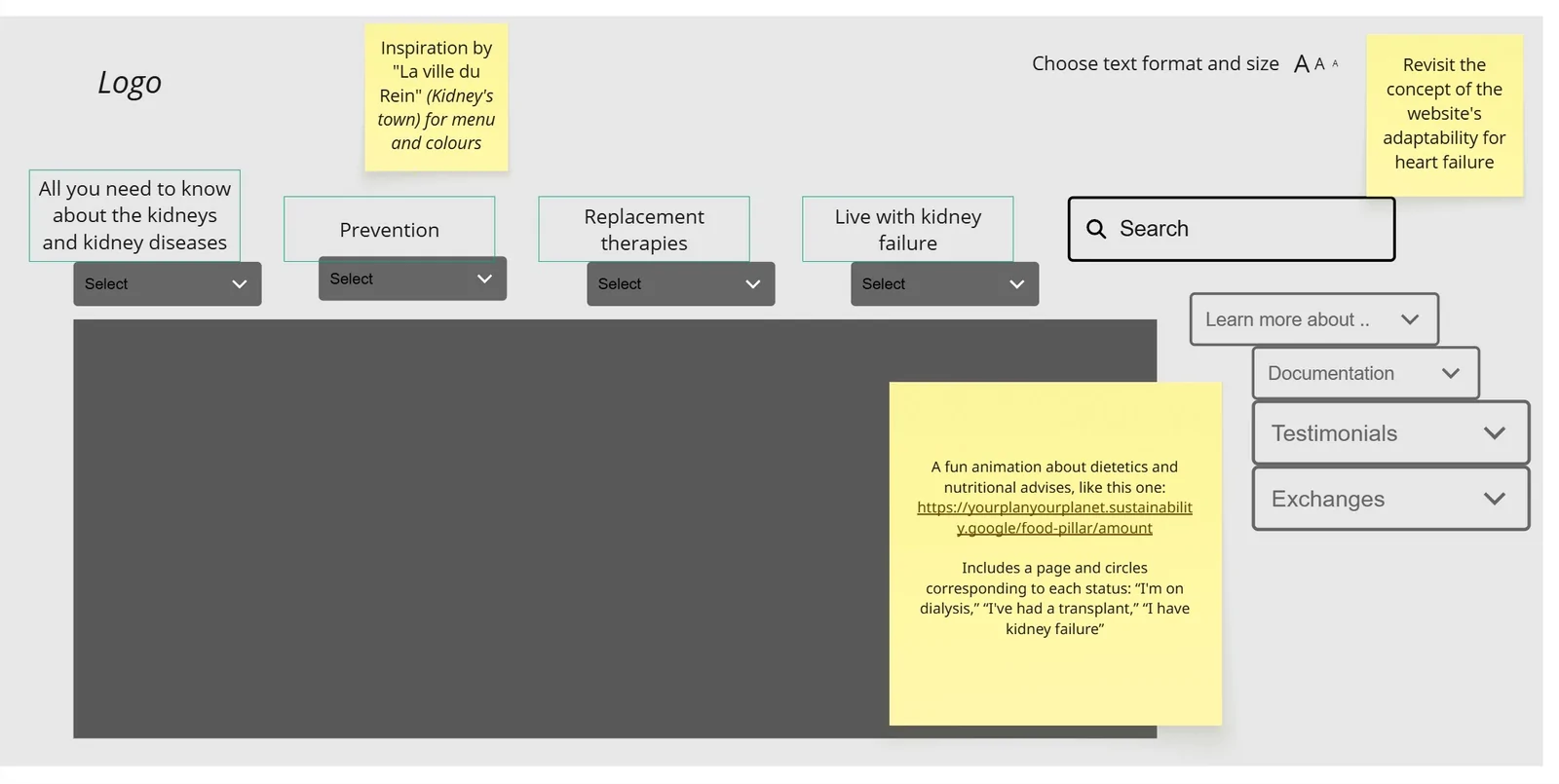
Accessibility-oriented mockup developed during formative co-design of the Renaclic CKD platform (France, 2021). CKD:

Early mockups were developed to translate co-design outputs into interface layouts (Figures 5 and 6).

Early interface layout integrating educational resources and navigation pathways defined by patients and health care professionals.

Prototype emphasizing simplified navigation, adaptable visual settings, and mobile-friendly design to address digital literacy considerations.

A low-fidelity prototype integrating educational content, symptom-tracking tools, and community features was developed.

### Data Analysis

Qualitative data were analyzed using thematic analysis. Card-sorting results informed the final content hierarchy. Analyses were conducted independently by two researchers, with discrepancies resolved by consensus.

## Results

### Benchmarking Findings

Review of 10 CKD-related platforms revealed fragmented educational content, limited information on home dialysis modalities and logistics, minimal psychosocial and lifestyle support, and low interactivity. Structured patient pathways and moderated peer-support spaces were generally absent.

### Interview Findings

Semi-structured interviews were conducted with 3 health care professionals and 5 patients (2 novice and 3 expert users). Five main themes emerged from interviews: (1) need for reliable and centralized information, (2) anxiety during care transitions, (3) demand for practical lifestyle guidance, (4) importance of positive and reassuring language, and (5) value of peer experience and testimonials. These themes directly informed platform content and design choices and were operationalized into more than 30 discrete thematic content items subsequently used during the card-sorting activity.

### Co-Design Outputs and Platform Prototype

Card-sorting exercises resulted in five core content categories: prevention, treatment options, kidney health, living with CKD, and further resources (as illustrated in Figure 4). Consolidated information structure organizing CKD-related content into five user-defined categories based on thematic analysis and participatory prioritization [10].

Interface discussions emphasized simplicity, adjustable font sizes, and guided navigation pathways. The *Renaclic* prototype integrates educational resources, symptom-tracking tools, and community-oriented features, optimized for mobile use.

## Discussion

### Principal Findings

This study describes the formative development of Renaclic through a structured user-centered design process involving patients and health care professionals in France. Benchmarking of 10 CKD-related platforms revealed fragmented content, limited coverage of practical home dialysis management, insufficient psychosocial resources, and minimal interactive features. Interviews with 3 health care professionals and 5 patients identified five core needs: reliable centralized information, support during care transitions, practical lifestyle guidance, reassuring language, and moderated peer interaction. These findings informed the information architecture and functional specifications of the Renaclic prototype.

### Interpretation and Comparison With Existing Literature

Consistent with prior research on digital CKD interventions, our findings confirm that availability of information alone is insufficient to ensure relevance and engagement [4,6,7]. Patients emphasized practical and experiential knowledge, particularly around dialysis logistics, work-life balance, and transplantation pathways—areas often underrepresented in institutional platforms.

The participatory design approach aligns with ISO 9241‐210 human-centered design principles [8] and recent literature advocating for structured co-design in digital health development [9,10]. By incorporating both novice and expert patients, this study highlights the heterogeneity of informational and psychosocial needs across CKD trajectories.

In addition, successful implementation of digital health tools depends not only on patient usability but also on professional acceptability and integration into clinical workflows [13]. Addressing both stakeholder perspectives at an early formative stage may facilitate future adoption and sustainability.

Digital health inequities remain a major challenge in chronic disease management [11,12]. Design decisions in Renaclic—such as simplified navigation, mobile adaptability, and visual accessibility features—were explicitly intended to mitigate digital literacy barriers identified during interviews.

### Limitations

This study has several limitations. First, the sample size was small (n=8), reflecting the formative and exploratory nature of the research. Although recruitment continued until no substantially new themes emerged within stakeholder categories, broader representation may have identified additional needs.

Second, data collection was conducted virtually, which may have limited participation among individuals with low digital access or literacy.

Third, quantitative usability testing and engagement metrics were not assessed at this stage. These aspects will require formal evaluation in subsequent implementation studies.

### Implications and Future Directions

This study contributes to the limited literature transparently reporting early-stage CKD digital co-design processes and provides a replicable methodological framework for formative development in chronic disease digital health. By combining benchmarking, thematic analysis, and structured co-design, Renaclic demonstrates how digital platforms can be grounded in both clinical accuracy and lived patient experience.

Future research will focus on usability testing, implementation in real-world nephrology settings, and evaluation of patient engagement, professional acceptability, and sustainability over time.

### Conclusions

This formative co-design study demonstrates how structured user-centered design can support the development of CKD digital platforms grounded in patient and professional needs. Renaclic provides a transparent and reproducible framework for early-stage digital health development. Future research will focus on usability testing and real-world implementation.

## Abbreviations

CKD: chronic kidney disease
HCP: health care professional
UCD: user-centered design
